# Associations Between Social Economic Determinants and Long-Term Outcomes of Critically Ill Patients

**DOI:** 10.1097/CCM.0000000000006587

**Published:** 2025-02-13

**Authors:** Dries van Sleeuwen, Floris A. van de Laar, Koen S. Simons, Daniëlle van Bommel, Dominique Burgers-Bonthuis, Julia Koeter, Laurens L.A. Bisschops, Inge Janssen, Thijs C.D. Rettig, Johannes G. van der Hoeven, Mark van den Boogaard, Marieke Zegers

**Affiliations:** 1 Department of Primary and Community Care, Radboud University Medical Center, Nijmegen, The Netherlands.; 2 Department of Intensive Care, Radboud University Medical Center, Nijmegen, The Netherlands.; 3 Department of Intensive Care Medicine, Jeroen Bosch Hospital’s Hertogenbosch, The Netherlands.; 4 Department of Intensive Care Medicine, Bernhoven Hospital Uden, The Netherlands.; 5 Department of Intensive Care Medicine, Rijnstate Hospital, Arnhem, The Netherlands.; 6 Department of Intensive Care Medicine, CWZ, Nijmegen, The Netherlands.; 7 Department of Intensive Care Medicine, Maasziekenhuis, Boxmeer, The Netherlands.; 8 Department of Anesthesiology, Intensive Care Medicine, and Pain Medicine, Amphia Hospital, Breda, The Netherlands.

**Keywords:** intensive care, patient-reported outcomes, social determinants of health, socioeconomic status

## Abstract

**OBJECTIVE:**

Differences in socioeconomic status (SES) may influence long-term physical, psychological, and cognitive health outcomes of ICU survivors. However, the relationship between SES and these three long-term health outcomes is rarely studied. The aim of this study was to investigate associations between SES and the occurrence of long-term outcomes 1-year post-ICU.

**DESIGN:**

Prospective cohort study.

**SETTING:**

Seven Dutch ICUs.

**PATIENTS:**

Patients 16 years old or older and admitted for greater than or equal to 12 hours to the ICU between July 2016 and March 2020 completed questionnaires, or relatives if patients could not complete them themselves, at ICU admission and 1 year after ICU admission.

**INTERVENTIONS:**

None.

**MEASUREMENTS AND MAIN RESULTS:**

Validated scales were used for the outcomes: physical problems (fatigue or ≥ 3 new physical symptoms), psychological problems (anxiety, depression, or post-traumatic stress), cognitive impairment, and a composite score. Occurrence of outcomes were calculated for: origin, education level, employment status, income, and household structure. Adjusted odds ratios (aORs) were calculated with covariates age, gender, admission type, severity-of-illness, and pre-ICU health status. Of the 6555 patients included, 3246 (49.5%) completed the questionnaires at admission and after 1 year. Low education level increased the risk of having health problems in the composite score 1-year post-ICU (aOR 1.84; 95% CI, 1.39–2.44; *p* < 0.001). Pre-ICU unemployment increased the risk of having physical problems (aOR 1.98; 95% CI, 1.31–3.01; *p* = 0.001). Migrants and low income was associated with more psychological problems (aOR 2.03; 95% CI, 1.25–3.24; *p* < 0.01; aOR 1.54; 95% CI, 1.10–2.16; *p* = 0.01, respectively), and unpaid work with less psychological (aOR 0.26; 95% CI, 0.08–0.73; *p* = 0.02) and cognitive (aOR 0.11; 95% CI, 0.01–0.59; *p* = 0.04) problems.

**CONCLUSIONS:**

Indicators of lower SES, including low education level, low income, unemployment and migrants were associated with an increased risk of post-ICU health problems. Gaining insight into the complex relationship between SES and long-term health problems is necessary to decrease disparities in healthcare.

**KEY POINTS**

**Question**: What are the associations between socioeconomic status (SES) and the occurrence of adverse long-term outcomes 1-year post-ICU?

**Findings**: In this prospective cohort study in seven Dutch ICUs, 6555 patients were included and 3246 completed questionnaires at admission and after 1 year. Former ICU patients with lower SES have a higher risk for health problems 1-year post-ICU.

**Meaning**: Gaining insight into the complex relationship between SES and long-term health problems is necessary to decrease disparities in healthcare and make ICU care and post-ICU care accessible and understandable for everyone.

Socioeconomic status (SES) is defined as one’s ability to access desired resources including human, materialistic, and social capital (1). SES is often determined by education, income, occupation, or a combination of all three (2). For decades, SES has been inextricably linked with health (3). Lower SES status is associated with a higher frequency of diseases and a higher disease burden, which could be due to several factors, such as poor access to healthcare and expensive healthcare costs for people with low incomes (4–7). In The Netherlands, citizens with low education levels are predicted to die 4 years earlier and live 15 years in poorer health compared with higher-educated citizens (8). These differences in health between population groups might even increase further, as became extremely visible during the COVID-19 pandemic (9, 10). This shows that differences in SES give rise to ongoing healthcare inequality and therefore may lead to new or worsening health problems in certain population groups.

This also applies to ICU patients, as previous studies show that a lower socioeconomic position is associated with higher mortality rates after critical illness (11). Lower SES position is also associated with impaired health-related quality of life (QoL) 6 months after critical illness (12). On the contrary, higher social integration (13) and social support (14) are associated with improved QoL after critical illness supporting the hypothesis that social relationships can mitigate negative effects of illness.

Although more patients survive ICU treatment due to advances in critical care medicine (15), the number of ICU survivors who experience long-lasting health problems increases. These problems impact work, daily functioning, and QoL (15–18). However, most previous ICU studies have focused on survivorship, and research on the importance of preadmission patient characteristics on recovery is growing (19). However, the association of SES with long-term outcomes and the level of functioning in the daily life of patients after critical illness remains unknown. The aim of the present study was to explore the association of SES (education level, income and employment status, origin and household structure) on long-term outcomes after ICU treatment.

## MATERIALS AND METHODS

### Study Design

Data for these studies was obtained from the ongoing prospective multicenter cohort MONITOR-IC study (www.clinicaltrials.gov identifier NCT 03246334). The MONITOR-IC study was approved by the ethics committee of the Radboud University Medical Center, Committee on Research Involving Human Subjects, region Arnhem-Nijmegen, The Netherlands (2016-2724) on August 23, 2016, and conducted in accordance with the declaration of Helsinki (20).

### Study Population

In the MONITOR-IC study, data of ICU patients 16 years and older and admitted for at least 12 hours to one of the seven participating hospitals in The Netherlands were collected. ICU patients (medical, elective surgical, and emergency surgical) admitted between July 2016 and March 2020 (pre-COVID-19) were included in the present study. Patients were excluded if they had died before ICU discharge or if they had a life expectancy of less than 48 hours, were not registered in The Netherlands, or could not read or speak the Dutch language.

### Data Collection

A questionnaire about the patient’s health status was completed both before ICU admission (baseline) and 1 year after ICU admission, either by the patients themselves or, if they were unable, by their relatives. Elective surgical patients received the baseline questionnaire at the preoperative outpatient clinic and completed the questionnaire a few days before their ICU admission. For medical and emergency surgical patients this was not possible and they therefore received the baseline questionnaire while in the ICU. These patients, or their relatives, were then asked to retrospectively rate the patients’ health status before ICU admission. Questionnaires were the same, regardless of the patient’s admission type and gave clear instructions for surrogates filling in the questionnaire. For the baseline measurement, a reminder was sent after 4 weeks and a reminder by telephone was provided 2 weeks later if necessary. For the 1-year questionnaire, reminders were sent after 2 and 4 weeks. Patient record data were collected in the first 24 hours of the ICU admission (21).

SES was assessed by the following variables obtained from the baseline questionnaire: origin, education level, employment situation, income, and household structure. Origin was categorized according to the new definition of Statistics Netherlands (CBS) as: country of origin: The Netherlands (person and both parents were born in The Netherlands), child of migrant(s) (person born in The Netherlands, but parent(s) born abroad) and migrant (person is born abroad) (22). Education level was categorized according to highest finished level of education as: low (unfinished primary education, primary education, lower-level secondary education), middle (intermediate general secondary education, secondary vocational education, pre-university education), or high (high professional education or academic education). Employment situation before ICU admission was categorized as: paid job, unemployed or social benefit, unpaid job, and retired. If a patient reported taking care of the household full-time or received a student grant, the patient was assigned to the group of unpaid jobs. Individual monthly net income was defined as: low (< 1700 euros), middle (1700–2700 euros), and high (> 2700 euros) according to previous research by The Netherlands Institute for Health Services Research (23). The household structure was categorized into five categories: living alone, living together with a partner without children, living together with a partner and children, living together with someone other than a partner, and living in a healthcare facility. Patients living alone or together with their partners within a nursing home were assigned to the group nursing home.

### Outcomes

Physical problems at baseline were objectified as extreme fatigue defined by a score of greater than or equal to 37 on the Checklist Individual Strength—fatigue subscale (CIS-8) (24, 25). Physical problems after 1 year were defined as extreme fatigue, or the presence of greater than or equal to three new physical problems as a consequence of ICU treatment, which were objectified by a list of 30 symptoms and rated as present if greater than or equal to three symptoms were moderate or severe. Psychological problems measured at baseline were anxiety or depression defined by a score of greater than or equal to 8 on the corresponding subscales of the Hospital Anxiety and Depression Scale (HADS) (26, 27). Measured psychological problems after 1 year were also anxiety and depression added with measurements of symptoms of post-traumatic stress disorder (PTSD). PTSD symptoms were defined by a mean of all questions greater than or equal to 1.75 on the Impact of Event Scale-6 (i.e., S-6) (28, 29). Cognitive impairment at baseline and after 1 year was determined with a score of greater than or equal to 43 on the abbreviated Cognitive Failure Questionnaire (CFQ-14) (30). Finally, a composite score of all physical, psychological, and cognitive problems was created. Patients were categorized as positive for the composite score if they had one or more positive scores (health problems) in the physical, psychological, and/or cognitive domains.

### Statistical Analysis

Baseline characteristics were presented as means with sds for normally distributed continuous variables, or medians with first and third quartiles (expressed in interquartile ranges [IQRs]) for not-normally distributed continuous variables, and counts with percentages for categorical variables. Patients who completed both baseline and the 1-year questionnaire were included in the analysis. Characteristics were compared between complete cases and non-responders (only the baseline questionnaire was completed) using the independent-sample *t* test, Mann-Whitney *U* test, or chi-square test. Missing values in the CIS-8 and HADS were imputed using the half rule, meaning that these values were replaced with the mean of the answered items in the subscale, if at least half of that subscale was answered (31). Missing values in the IES-R were replaced with the individual mean, provided that 75% of the items were completed. If a patient’s country of birth was missing and one parent or both parents were born abroad, the patient was assigned to the group child of migrant(s). If a patient’s employment status was missing and the patient reported working for greater than or equal to 12 hours a week before ICU admission, the patient was assigned to the group of paid jobs according to the definition of Statistics Netherlands. If a patient’s employment status was missing, and his/her work week used to be less than 12 hours (or also missing), and his/her age was greater than or equal to 67 years (Dutch state pension age), the patient was assigned to the retired group.

Outcomes were dichotomized using the cutoffs specified above. To explore SES risk factors associated with physical, psychological, and cognitive health problems and the composite score 1 year after ICU admission, multivariable logistic regression analyses were conducted, with age, gender, admission type, and severity-of-illness score (Acute Physiology and Chronic Health Evaluation IV [APACHE IV]) serving as covariates. The model with the outcome of physical problems was adjusted for fatigue at baseline. The model for psychological problems was adjusted for anxiety and depression at baseline. The model for cognitive problems was adjusted for cognitive functioning at baseline. These analyses were also performed separately with symptoms of fatigue, anxiety, depression, PTSD, cognitive impairment, and new physical complaints as outcomes. Here, the models with the outcome fatigue, anxiety, and depression were adjusted for the baseline scores of respectively fatigue, anxiety, and depression. Generalized Variance Inflation Factors ($GVIF{}^{\frac{1}{2DegreesofFreedom}}$) (32) were calculated for all variables of the developed models depending on the outcome to evaluate the amount of multicollinearity. Adjusted odds ratios (aORs) and 95% CIs were calculated. All analyses were performed using R software, version 4.1.3 (R Foundation for Statistical Computing) (packages tidyverse, haven, dplyr, magrittr, foreign, labeled, stats, pastecs, crosstable, car, oddsratio, and ggplot2).

### Correlation

No significant collinearity was found with GVIF values less than 2 for all variables. In addition, to gain more insight into the variables’ distribution among themselves, Pearson’s chi-square tests and Fisher exact tests were calculated for these variables (**Supplemental 1**, **Tables E1–E3**, http://links.lww.com/CCM/H674).

## RESULTS

### Study Population

**Figure 1.**
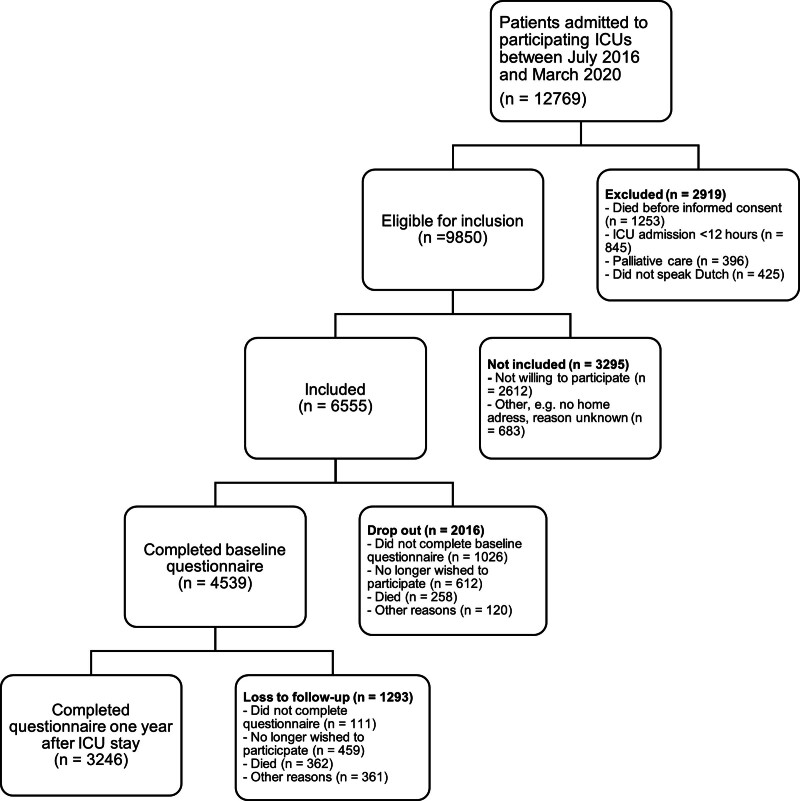
Flowchart of participants.

In total, 3246 patients filled in the questionnaire at baseline and the questionnaire 1 year after ICU admission (**Fig. 1**). 81.7% of the baseline questionnaires were filled in by patients (and 88.3% of the 1-yr questionnaires), the remainder by or together with proxies. The mean age was 63.3 years (sd 13.3) and 66.0% were male. The mean APACHE IV score was 54.4 (sd 20.8) (**Table 1**; and **Supplemental 2**, http://links.lww.com/CCM/H674). Characteristics of patients who only completed the baseline questionnaire at ICU admission are shown in **Supplemental 3** (http://links.lww.com/CCM/H674).

**TABLE 1.** Characteristics of the Included Patients

Patient Characteristics	All Patients (*n* = 3246)	Missing, *n/N* (%)
Age, yr, mean (sd)	63.3 (13.3)	64 (2.0)
16–39 yr, *n*/*N* (%)	191 (5.9)	
40–64 yr, *n*/*N* (%)	1263 (38.9)	
65–79 yr, *n*/*N* (%)	1547 (47.7)	
≥ 80 yr, *n*/*N* (%)	181 (5.6)	
Gender, *n*/*N* (%)		1 (0.03)
Male	2143 (66.0)	
Female	1102 (33.9)	
Admission type, *n*/*N* (%)		67 (2.1)
Medical	980 (30.2)	
Emergency surgery	384 (11.8)	
Elective surgery	1815 (55.9)	
Length of ICU stay, *n*/*N* (%)		64 (2.0)
< 2 d	2223 (68.5)	
≥ 2 d	959 (29.5)	
Acute Physiology and Chronic Health Evaluation IV score, mean (sd)	54.4 (20.8)	64 (2.0)
Origin, *n*/*N* (%)		179 (5.5)
Country of origin: The Netherlands	2777 (85.6)	
Child of migrant(s)	170 (5.2)	
Migrant	120 (3.7)	
Education level, *n*/*N* (%)		53 (1.6)
Low	966 (29.8)	
Middle	1384 (42.6)	
High	843 (26.0)	
Employment status, *n*/*N* (%)		379 (11.7)
Employed^a^	1311 (40.4)	
Unemployed (or social benefit)	139 (4.3)	
Non-paid job^b^	50 (1.5)	
Retired	1367 (42.1)	
Net month income (euro), *n*/*N* (%)		391 (12.0)
< 1700	1359 (41.9)	
1700–2700	957 (29.5)	
> 2700	539 (16.6)	
Household structure, *n*/*N* (%)		60 (1.9)
Alone	537 (16.5)	
With partner without children	1981 (61.0)	
With partner and children	477 (14.7)	
Together with someone else^c^	162 (5.0)	
Nursing home	29 (0.9)	

a Paid work, own company, or living on one’s investments.

b Such as homemaker.

c Living together with someone else than partner, such as brother, parents, or child.

The extended version is shown in Supplemental 2 (http://links.lww.com/CCM/H674).

### Occurrence of Health Problems Post-ICU

One year after ICU admission, the prevalence of physical, psychological, or cognitive problems was 44.0%, 31.5%, and 10.8%, respectively. The occurrence rate of having one health problem (composite score) was 51.9%. Occurrences of physical, psychological, and cognitive problems 1 year after ICU admission for patient groups of different social determinants are shown in **Supplemental 4** (http://links.lww.com/CCM/H674). The prevalence of health problems post-ICU among migrants was 61.9% vs. 53.3% among originally Dutch people; whereas this was the case for 65.2% among low-educated people and 43.7% among high-educated people. The prevalence of fatigue, new physical complaints, anxiety and depression symptoms, PTSD symptoms, and cognitive impairment is shown in **Supplemental 5** (http://links.lww.com/CCM/H674). A higher percentage of people with a low income reported fatigue 1 year after ICU admission (63.8%) compared with people with a high income (39.9%). Symptoms of depression were reported by 32.5% among migrants, vs. 22.4% among originally Dutch patients.

### Associations of Social Determinants and Health Problems Post-ICU

Low (vs. high) educated ICU survivors had an increased risk of having physical (aOR 1.66; 95% CI, 1.28–2.16; *p* < 0.001) and psychological (aOR 1.77; 95% CI, 1.32–2.38; *p* < 0.001) problems (**Table 2**; and **Supplemental 6**, http://links.lww.com/CCM/H674). Unemployed ICU survivors had an increased risk of having physical problems (aOR 1.98; 95% CI, 1.31–3.01; *p* = 0.001) vs. employed patients. Migrants and low-income individuals had an increased risk of having psychological problems post-ICU (aOR 2.03; 95% CI, 1.25–3.24; *p* < 0.01; aOR 1.54; 95% CI, 1.10–2.16; *p* = 0.01, respectively) versus originally Dutch patients and high-income individuals. Having a non-paid job (vs. employment) was associated with less psychological (aOR 0.26; 95% CI, 0.08–0.73; *p* = 0.02) and cognitive (aOR 0.11; 95% CI, 0.01–0.59; *p* = 0.04) problems. Household structure did not show any significant association with long-term health problems.

**TABLE 2.** Associations Between Social Determinants of Health and Health Problems 1 Year After ICU Admission (*n* = 3246)

Social Determinants, *N* (%)	Physical Problems		Psychological Problems		Cognitive Problems	
Origin	aOR (95% CI)	*p*	aOR (95% CI)	*p*	aOR (95% CI)	*p*
Country of origin: The Netherlands	Reference		Reference		Reference	
Child of migrant(s)	0.89 (0.59–1.32)	0.56	0.80 (0.50–1.25)	0.33	0.74 (0.35–1.43)	0.40
Migrant	1.46 (0.93–2.28)	0.10	**2.03 (1.25–3.24**)	**< 0.01**	1.18 (0.54–2.32)	0.66
Education level						
High	Reference		Reference		Reference	
Middle	**1.26 (1.00–1.57**)	**0.05**	1.10 (0.84–1.43)	0.49	1.28 (0.86–1.93)	0.22
Low	**1.66 (1.28–2.16**)	**< 0.001**	**1.77 (1.32–2.38**)	**< 0.001**	1.43 (0.91–2.26)	0.12
Employment status						
Employed^a^	Reference		Reference		Reference	
Unemployed (or social benefit)	**1.98 (1.31–3.01**)	**0.001**	1.18 (0.74–1.84)	0.48	1.40 (0.74–2.51)	0.28
Non-paid job^b^	0.63 (0.27–1.42)	0.28	**0.26 (0.08–0.73**)	**0.02**	**0.11 (0.01–0.59**)	**0.04**
Retired	1.13 (0.88–1.45)	0.35	1.20 (0.90–1.61)	0.21	0.80 (0.51–1.25)	0.32
Net income (euro)						
> 2700	Reference		Reference		Reference	
1700–2700	1.10 (0.85–1.42)	0.49	**1.43 (1.05–1.95**)	**0.02**	**1.79 (1.10–3.00**)	**0.02**
< 1700	1.10 (0.83–1.47)	0.51	**1.54 (1.10–2.16**)	**0.01**	1.38 (0.80–2.43)	0.25
Household structure						
Alone	Reference		Reference		Reference	
With partner without children	0.98 (0.77–1.25)	0.86	0.82 (0.63–1.08)	0.16	0.82 (0.55–1.24)	0.33
With partner and children	1.03 (0.74–1.43)	0.87	0.86 (0.59–1.25)	0.44	1.17 (0.70–1.97)	0.55
Together with someone else^c^	1.28 (0.77–2.14)	0.34	1.29 (0.73–2.25)	0.37	1.74 (0.83–3.53)	0.13
Nursing home	0.80 (0.28–2.35)	0.68	0.57 (0.18–1.70)	0.33	1.72 (0.35–6.34)	0.45

aOR = adjusted odds ratio.

a Paid work, own company, or living on one’s investments.

b Such as homemaker.

c Living together with someone else than partner, such as brother, parents or child.

Boldface font indicates statistically significant difference (*p* < 0.05). Adjusted for: age, gender, admission type, Acute Physiology and Chronic Health Evaluation IV score, and baseline score of the health variable (not applicable for new physical complaints and posttraumatic stress disorder).

The extended version is shown in Supplemental 6 (http://links.lww.com/CCM/H674).

Associations between social determinants and fatigue, new physical complaints and symptoms of anxiety, depression and PTSD symptoms are shown in **Supplemental 7** (http://links.lww.com/CCM/H674).

## DISCUSSION

In this large multicenter prospective cohort study, we found that education level, income and employment status were associated with long-term health outcomes after ICU admission. Low (vs. high) education level was associated with an increased risk of physical and psychological health problems 1 year after ICU. Migrants (vs. originally Dutch) and low (vs. high)-income individuals had an increased risk for psychological health problems after ICU admission. Pre-ICU unemployment was also associated with an increased risk for physical problems and having a non-paid job with a decreased risk for psychological and cognitive problems 1 year after ICU admission vs. employment. Possible explanations could be that having a paid job often comes with responsibilities, which can contribute to stress during recovery from critical illness. Having a non-paid job could also lead to more social engagement and flexibility which can contribute to a better health experience. No association was found between post-ICU health problems and patients’ household structure.

### Comparison With Literature

Most previous studies examined the association between SES and mortality. There are some studies with long-term health outcomes (33). In a French study, no differences were found between socioeconomically nondeprived and deprived ICU survivors for psychological health problems 1 year after ICU discharge, taking into account patients’ income, education level, and employment status (34). This is not in line with the present study, where these areas of SES were associated with psychological problems. One U.S. study found that non-White and poorly educated patients with shock and respiratory failure experienced more long-term post-ICU cognitive impairment (35). Another U.S. study reported that more years of education was associated with a greater risk of having no post-ICU health problems (36). This is in line with the present study and may be attributed to systemic barriers such as racial discrimination, socioeconomic deprivation, and inequities in access to education and healthcare, which collectively limit health literacy and prevent many individuals from fully benefiting from treatments and information available to manage or prevent illness (37). Also, the quality of care and communication between healthcare professionals and patients from ethnic minorities may differ (38). Another U.S. study found that community-dwelling older patients eligible for government healthcare coverage experienced more functional decline in daily living and mobility and cognition after ICU discharge, but did not find any associations with symptoms of depression and anxiety (39). This is in line with the present study, although the increased risk of psychological and cognitive health problems was not significant. This could be explained by a larger sample size of the present study and virtually including all ICU survivors, regardless of their admission diagnosis or SES status. Differences could also be explained by a different social welfare system in The Netherlands. As the Dutch safety net creates relatively equal access to healthcare, the results of the present study might be an underestimation compared with countries with a poorer safety net. This might even lead to difficulties in conducting this research in countries with unequal access to healthcare, because of differences in quality of healthcare, independently of SES.

### Limitations

There are some limitations that need to be addressed. First, there might be a selection bias because patients who could not read or speak the Dutch language were excluded. Also, a non-response bias is likely because the individuals included in the analysis had a significantly better pre-ICU health status (16) and indicators of higher SES (e.g., higher income and education level) than non-responders (Supplemental 3, http://links.lww.com/CCM/H674). As a result, the findings of this study may underestimate the associations between SES and the occurrence of long-term health outcomes. Second, some questionnaires were filled in by proxies in case patients were unable to do it themselves. This could be criticized because their perception could differ from the patient (40, 41). However, previous baseline functioning assessment was found to be quite like-minded (42–44). Third, as SES is complex to define due to multiple determinants, variables such as sexual identity, skin color, access to healthcare, and neighborhood SES were not taken into account (45). However, health insurance is obligated for all Dutch citizens and the Dutch benefit system is designed to support people on low incomes. The usage of patients’ address data for additional SES calculations is prohibited by Dutch privacy legislation. Furthermore, variables of SES were only assessed individually in this study. Several instruments exist to measure SES as one total score, but this can be difficult as an SES measure may have a different meaning in different social groups or countries (46). Fourth, the results of this study may not be generalizable to other (low-income) countries as income, welfare system, and population heterogeneity may be different. Fifth, net income is calculated by individual income, so family income was not taken into account in this study.

### Implications

Based on the results of the present study, especially low education level seems to be an important risk factor for having long-term functional health problems after critical illness. Efforts in better communication tailored to the capacities of patients and their relatives, and information that is easy to understand for all people is necessary to prevent difference in health outcomes. This also requires considering the broader living environment. Differences in infrastructure, air quality, and caregiving responsibilities can impact individuals’ ability to pursue education and employment, influencing income and, consequently, access to healthcare (7). Furthermore, SES is a complex and multifactorial phenomenon, which makes it difficult to integrate its multiple domains as a whole (e.g., poorly educated people do not necessarily have to be on low income). Therefore, the results of this study highlight the need for personalized care, as well as community-based interventions and enhanced social services. This also applies to post-ICU care, which should be focused on the specific needs and capacities of patients and the problems they experience (47). Hospital professionals should collaborate with primary care professionals because the latter are often well aware of patients’ SES status. The findings of the present study can also help in selecting ICU survivors who have the highest risk of having post-ICU health problems at an early stage to prevent or mitigate post-ICU health problems. To further implement awareness for differences in SES in post-ICU care or prevention strategies, more uniform research is needed. SES classification tools exist, but the major obstacle could be that most SES outcomes are country-specific (48, 49). To further assess and compare patients’ SES status worldwide, international SES classification tools should be developed.

## CONCLUSIONS

This prospective cohort study showed associations between several social determinants of health with adverse outcomes 1 year after ICU admission. Indicators of lower SES, such as low education level, low income, unemployment, and migrants were associated with an increased risk of having post-ICU health problems. Gaining insight into the complex relationship of SES and long-term health problems is necessary to identify targets for policy implications that can decrease disparities in healthcare, making ICU care and post-ICU care accessible and understandable for everyone.

## ACKNOWLEDGMENTS

The authors thank all of the patients and their relatives for their participation in this study. In addition, they thank the ICU staff of the Canisius Wilhelmina Hospital (Nijmegen), Jeroen Bosch Hospital (’s-Hertogenbosch), Rijnstate Hospital (Arnhem), Bernhoven Hospital (Uden), Maasziekenhuis (Boxmeer), Amphia Hospital (Breda) and Radboud University Medical Center (Nijmegen) for their contribution to this study. They also thank (in alphabetical order) Ed van Mackelenberg, Juliette Cruijsberg, Nicky Eijkenboom-Wattel, Rachel Quibell-Melssen, Sanne Schröduer, and Sjef van der Velde for their advice and support in performing this study. Furthermore, they thank the national foundation Family and Patient Centered Intensive Care, and patient organization IC Connect for ICU survivors and their family members for their close cooperation.

## Supplementary Material
