# Supplementary material for: Associations Between Social Economic Determinants and Long-Term Outcomes of Critically Ill Patients
Source: Crit Care Med. 2025 Feb 13;53(4):e783–93. doi: 10.1097/CCM.0000000000006587 (PMC11952688; doi:10.1097/CCM.0000000000006587)
Supplement: Supplementary file 1 [file ccm-53-e783-s001.docx]

**Supplementary files**

Table of contents

[Supplemental 1. 3](#_Toc179128132)

[Table E1. Distribution by education level 4](#_Toc179128133)

[Table E2. Distribution by net month income 5](#_Toc179128134)

[Table E3. Distribution by employment status 6](#_Toc179128135)

[Supplemental 2. 7](#_Toc179128136)

[Supplemental 3. 9](#_Toc179128137)

[Supplemental 4. 11](#_Toc179128138)

[Supplemental 5. 13](#_Toc179128139)

[Supplemental 6. 16](#_Toc179128140)

[Supplemental 7. 18](#_Toc179128141)

# Supplemental 1.

Associations between variables.

Additional tests were performed to calculate the interaction of socioeconomic status (SES) variables used in the main analysis.

# Table E1. Distribution by education level


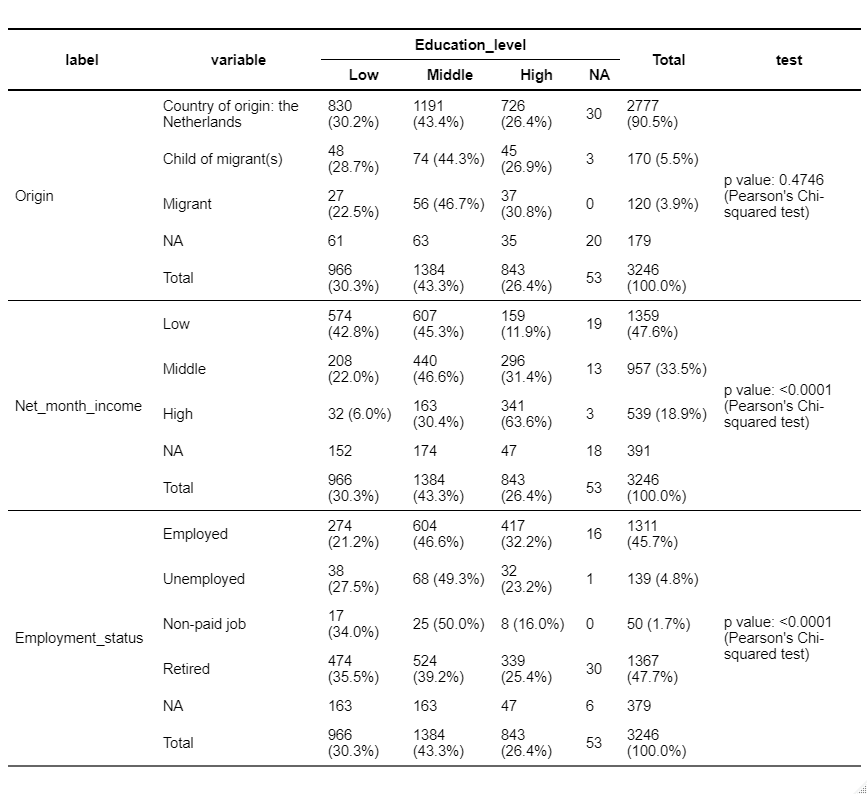


*Distribution of origin, net month income and employment status by education level. Distributions are showed in percentages (%). X2 Pearson chi-square test are calculated.*

# Table E2. Distribution by net month income


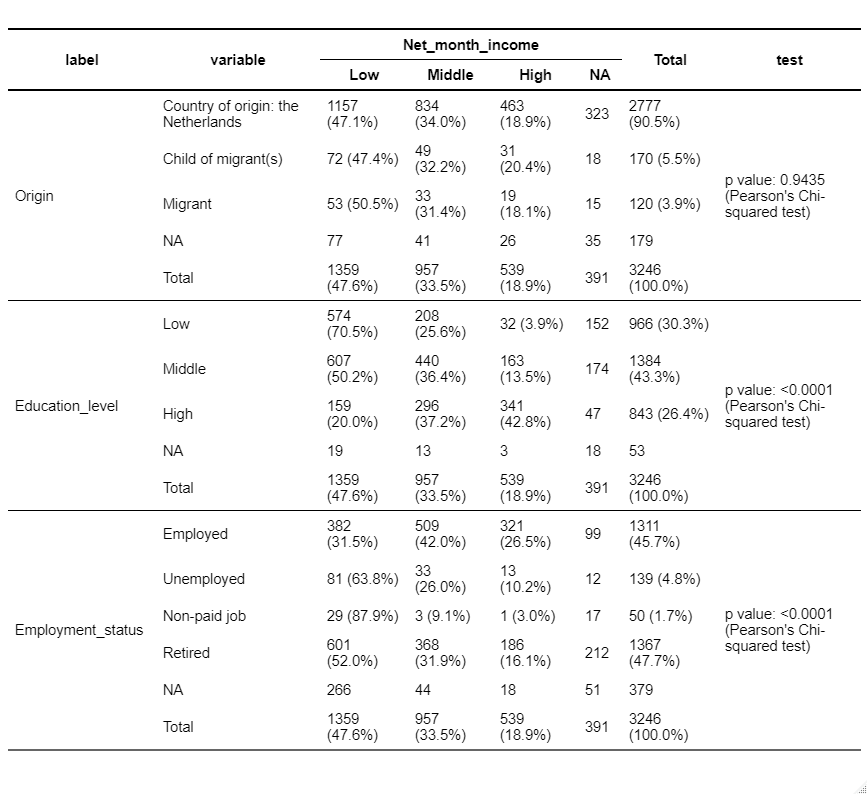


*Distribution of origin, education level and employment status by net month income. Distributions are showed in percentages (%). X2 Pearson chi-square test are calculated.*

# Table E3. Distribution by employment status

*
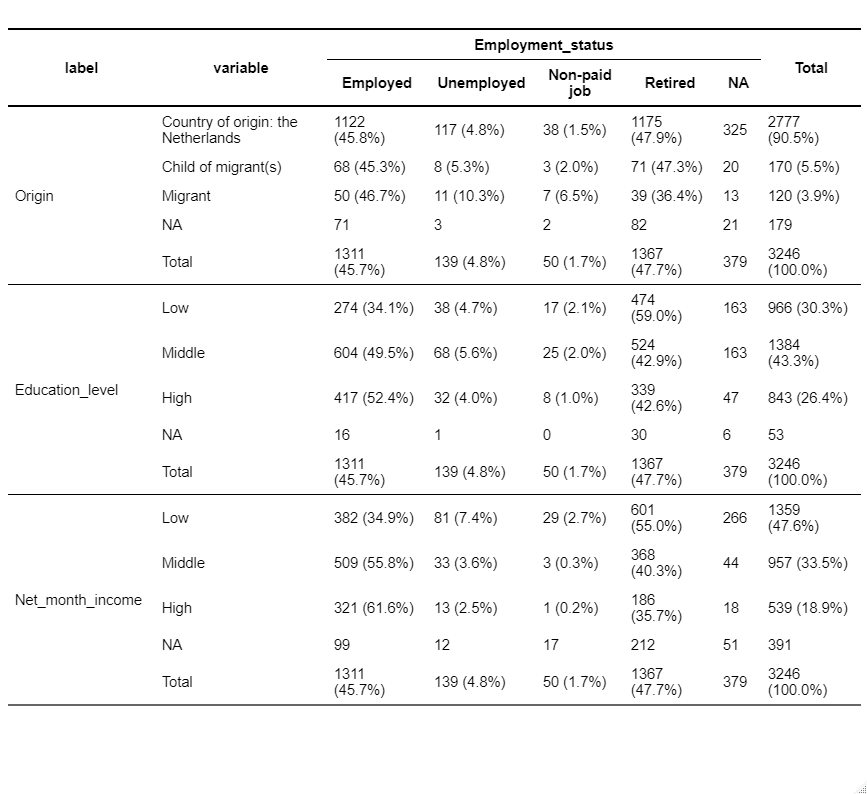
*

*p-value: 0.003
(Fisher’s exact test)*

*Distribution of origin, education level and net month income by employment status. Distributions are showed in percentages (%).*

# Supplemental 2.

Characteristics of the included patients (extended version of Table 1 in the main text).

| Patient characteristics | | All patients (n = 3246) | |
| --- | --- | --- | --- |
| Age, years, mean (SD) | | 63.3 | (13.3) |
|  | *Missing* | 64 | (2.0) |
| Age categories, years, – n/N (%) | |  |  |
|  | 16–39 yr – n/N (%) | 191 | (5.9) |
|  | 40–64 yr – n/N (%) | 1263 | (38.9) |
|  | 65–79 yr – n/N (%) | 1547 | (47.7) |
|  | ⩾80 yr – n/N (%) | 181 | (5.6) |
| Gender – n/N (%) | |  |  |
|  | Male | 2143 | (66.0) |
|  | Female | 1102 | (33.9) |
|  | *Missing* | 1 | (0.03) |
| Admission type – n/N (%) | |  |  |
|  | Medical | 980 | (30.2) |
|  | Emergency surgery | 384 | (11.8) |
|  | Elective surgery | 1815 | (55.9) |
|  | *Missing* | 67 | (2.1) |
| Length of ICU stay – n/N (%) | |  |  |
|  | <2 days | 2223 | (68.5) |
|  | ≥ 2 days | 959 | (29.5) |
|  | *Missing* | 64 | (2.0) |
| APACHE IV score - mean (SD) | | 54.4 | (20.8) |
|  | *Missing* | 64 | (2.0) |
| APACHE IV diagnosis groups – n/N (%) | |  |  |
|  | Cardiovascular | 530 | (16.3) |
|  | Cardiovascular surgery | 1315 | (40.5) |
|  | Neurological | 176 | (5.4) |
|  | Pulmonary | 207 | (6.4) |
|  | Trauma | 180 | (5.6) |
|  | Other | 838 | (25.8) |
| Origin – n/N (%) | |  |  |
|  | Country of origin: the Netherlands | 2777 | (85.6) |
|  | Child of migrant(s) | 170 | (5.2) |
|  | Migrant | 120 | (3.7) |
|  | *Missing* | 179 | (5.5) |
| Education level – n/N (%) | |  |  |
|  | Low | 966 | (29.8) |
|  | Middle | 1384 | (42.6) |
|  | High | 843 | (26.0) |
|  | *Missing* | 53 | (1.6) |
| Employment status– n/N (%) | |  |  |
|  | Employed ^*^ | 1311 | (40.4) |
|  | Unemployed (or social benefit) | 139 | (4.3) |
|  | Non-paid job^†^ | 50 | (1.5) |
|  | Retired | 1367 | (42.1) |
|  | *Missing* | 379 | (11.7) |
| Net month income (euro) - n/N (%) | |  |  |
|  | <1700 | 1359 | (41.9) |
|  | 1700-2700 | 957 | (29.5) |
|  | > 2700 | 539 | (16.6) |
|  | *Missing* | 391 | (12.0) |
| Household structure - n/N (%) | |  |  |
|  | Alone | 537 | (16.5) |
|  | With partner without children | 1981 | (61.0) |
|  | With partner and children | 477 | (14.7) |
|  | Together with someone else^‡^ | 162 | (5.0) |
|  | Nursing home | 29 | (0.9) |
|  | *Missing* | 60 | (1.9) |

Definition of abbreviations: APACHE IV = Acute Physiology and Chronic Health Evaluation IV; SD = standard deviation

* Paid work, own company or living on one’s investments

† Such as homemaker

‡ Living together with someone else than partner, such as brother, parents or child

# Supplemental 3.

Characteristics non-responders (n = 1293) who only completed the baseline questionnaire at ICU admission, but not after one year.

| Patient characteristics | | Non-responders (n = 1293) | | Complete cases (n = 3246) | | p-value |
| --- | --- | --- | --- | --- | --- | --- |
| Age, years, mean (SD) | | 62.1 | (15.5) | 63.3 | (13.3) | .02* |
|  | *Missing* | 28 | (2.2) | 64 | (2.0) |  |
| Gender – n/N (%) | |  |  |  |  | <.001* |
|  | Male | 772 | (59.7) | 2143 | (66.0) |  |
|  | Female | 520 | (40.2) | 1102 | (33.9) |  |
|  | *Missing* | 1 | (.08) | 1 | (0.03) |  |
| Admission type – n/N (%) | |  |  |  |  | <.001* |
|  | Medical | 487 | (37.7) | 980 | (30.2) |  |
|  | Emergency surgery | 149 | (11.5) | 384 | (11.8) |  |
|  | Elective surgery | 627 | (48.5) | 1815 | (55.9) |  |
|  | *Missing* | 30 | (2.3) | 67 | (2.1) |  |
| Length of ICU stay – n/N (%) | |  |  |  |  | <.001* |
|  | <2 days | 797 | (61.6) | 2223 | (68.5) |  |
|  | ≥ 2 days | 468 | (36.2) | 959 | (29.5) |  |
|  | *Missing* | 28 | (2.2) | 64 | (2.0) |  |
| APACHE IV score - mean (SD) | | 56.1 | (24.7) | 54.4 | (20.8) | .55 |
|  | *Missing* | 28 | (2.2) | 64 | (2.0) |  |
| Origin – n/N (%) | |  |  |  |  | <.002* |
|  | Country of origin: the Netherlands | 1036 | (80.1) | 2777 | (85.6) |  |
|  | Child of migrant(s) | 78 | (6.0) | 170 | (5.2) |  |
|  | Migrant | 74 | (5.7) | 120 | (3.7) |  |
|  | *Missing* | 105 | (8.1) | 179 | (5.5) |  |
| Education level – n/N (%) | |  |  |  |  | <.001* |
|  | Low | 535 | (41.4) | 966 | (29.8) |  |
|  | Middle | 528 | (40.8) | 1384 | (42.6) |  |
|  | High | 207 | (16.0) | 843 | (26.0) |  |
|  | *Missing* | 23 | (1.8) | 53 | (1.6) |  |
| Employment status– n/N (%) | |  |  |  |  | <.001* |
|  | Employed ^*^ | 438 | (34.0) | 1311 | (40.4) |  |
|  | Unemployed (or social benefit) | 86 | (6.7) | 139 | (4.3) |  |
|  | Non-paid job^†^ | 24 | (1.9) | 50 | (1.5) |  |
|  | Retired | 532 | (41.1) | 1367 | (42.1) |  |
|  | *Missing* | 213 | (16.5) | 379 | (11.7) |  |
| Net month income (euro) - n/N (%) | |  |  |  |  | <.001* |
|  | <1700 | 584 | (45.2) | 1359 | (41.9) |  |
|  | 1700-2700 | 329 | (25.4) | 957 | (29.5) |  |
|  | > 2700 | 114 | (8.8) | 539 | (16.6) |  |
|  | *Missing* | 266 | (20.6) | 391 | (12.0) |  |
| Household structure - n/N (%) | |  |  |  |  | <.001* |
|  | Alone | 261 | (20.2) | 537 | (16.5) |  |
|  | With partner without children | 668 | (51.7) | 1981 | (61.0) |  |
|  | With partner and children | 182 | (14.1) | 477 | (14.7) |  |
|  | Together with someone else^‡^ | 117 | (9.1) | 162 | (5.0) |  |
|  | Nursing home | 29 | (2.2) | 29 | (0.9) |  |
|  | *Missing* | 36 | (2.8) | 60 | (1.9) |  |

Definition of abbreviations: APACHE IV = Acute Physiology and Chronic Health Evaluation IV; SD = standard deviation

* Paid work, own company or living on one’s investments

† Such as homemaker

‡ Living together with someone else than partner, such as brother, parents or child

* Statistically significant difference (p <.05) between complete cases and loss to follow-up.

# Supplemental 4.

Occurrence rates of physical, psychological and cognitive health problems one year after ICU admission for different social determinants of health.

| Social determinants – N (%) | |  | Physical problems | | Psychological problems | | Cognitive problems | | Composite score^§^ | |
| --- | --- | --- | --- | --- | --- | --- | --- | --- | --- | --- |
| Origin | |  |  | |  | |  | |  | |
|  | Country of origin: the Netherlands | (n = 2777) | 1208 | (43.7%) | 859 | (31.2%) | 299 | (11.5%) | 1428 | (53.3%) |
|  | Child of migrant(s) | (n = 170) | 70 | (41.9%) | 51 | (30.9%) | 15 | (9.4%) | 83 | (50.3%) |
|  | Migrant | (n = 120) | 67 | (55.8%) | 53 | (44.5%) | 19 | (16.5%) | 73 | (61.9%) |
|  | *Missing* |  | 84 |  | 60 |  | 18 |  | 101 |  |
| Education level | |  |  |  |  |  |  |  |  |  |
|  | Low | (n = 966) | 494 | (51.6%) | 403 | (42.3%) | 130 | (14.5%) | 608 | (65.2%) |
|  | Middle | (n = 1384) | 608 | (44.3%) | 411 | (30.0%) | 156 | (11.9%) | 696 | (52.0%) |
|  | High | (n = 843) | 303 | (36.0%) | 193 | (23.1%) | 63 | (7.9%) | 355 | (43.7%) |
|  | *Missing* |  | 24 |  | 16 |  | 2 |  | 26 |  |
| Employment status | |  |  |  |  |  |  |  |  |  |
|  | Employed ^*^ | (n = 1311) | 522 | (40.0%) | 362 | (27.9%) | 144 | (11.5%) | 619 | (48.9%) |
|  | Unemployed (or social benefit) | (n = 139) | 86 | (62.3%) | 55 | (39.6%) | 22 | (17.2%) | 92 | (67.7%) |
|  | Non-paid job^†^ | (n = 50) | 18 | (36.0%) | 13 | (26.0%) | 4 | (8.3%) | 22 | (44.9%) |
|  | Retired | (n = 1367) | 587 | (43.3%) | 433 | (32.2%) | 118 | (9.3%) | 708 | (54.0%) |
|  | *Missing* |  | 216 |  | 160 |  | 63 |  | 244 |  |
| Net month income (euro) | |  |  |  |  |  |  |  |  |  |
|  | <1700 | (n = 1359) | 703 | (52.2%) | 522 | (38.9%) | 183 | (14.4%) | 825 | (62.6%) |
|  | 1700-2700 | (n = 957) | 387 | (40.5%) | 271 | (28.5%) | 104 | (11.4%) | 452 | (48.7%) |
|  | > 2700 | (n = 539) | 179 | (33.3%) | 104 | (19.5%) | 30 | (6.0%) | 210 | (41.0%) |
|  | *Missing* |  | 160 |  | 126 |  | 34 |  | 198 |  |
| Household structure | |  |  |  |  |  |  |  |  |  |
|  | Alone | (n = 537) | 260 | (49.1%) | 202 | (38.1%) | 57 | (11.5%) | 311 | (60.2%) |
|  | With partner without children | (n = 1981) | 845 | (42.9%) | 582 | (29.7%) | 188 | (10.1%) | 997 | (52.1%) |
|  | With partner and children | (n = 477) | 203 | (42.7%) | 147 | (31.0%) | 63 | (13.9%) | 231 | (50.2%) |
|  | Together with someone else^‡^ | (n = 162) | 75 | (46.3%) | 59 | (36.7%) | 34 | (22.1%) | 91 | (57.6%) |
|  | Nursing home | (n = 29) | 18 | (62.1%) | 9 | (32.1%) | 5 | (19.2%) | 22 | (78.6%) |
|  | *Missing* |  | 28 |  | 24 |  | 4 |  | 33 |  |
| All patients (n = 3246) | | | (n = 1429) | | (n = 1023) | | (n = 351) | | (n = 1685) | |

Definition of abbreviations:

* Paid work, own company or living on one’s investments

† Such as homemaker

‡ Living together with someone else than partner, e.g., brother, parents or child

^§^ Composite score of physical, psychological and cognitive health problems

# Supplemental 5.

Occurrence of physical, psychological and cognitive problems at baseline and one year follow-up after ICU admission.

|  | | | **Physical** | | | **Psychological** | | | | | **Cognitive** | |
| --- | --- | --- | --- | --- | --- | --- | --- | --- | --- | --- | --- | --- |
|  |  |  | Fatigue | | New physical complaints | Anxiety | | Depression | | PTSD | Cognitive impairment | |
| **Social determinants – N (%)** | | | *ICU admission* | 1 year | 1 year | *ICU admission* | 1 year | *ICU admission* | 1 year | 1 year | *ICU admission* | 1 year |
| *All patients (n = 3246)* | | | (n = 2015) | (n = 1778) | (n = 1081) | (n = 785) | (n = 689) | (n = 729) | (n = 735) | (n = 198) | (n = 168 ) | (n = 351) |
| **Origin** | | |  |  |  |  |  |  |  |  |  |  |
|  | Country of origin: the Netherlands | (n = 2777) | 1713 (62.1) | 1515 (54.8) | 907 (32.7) | 645 (23.3) | 580 (21.0) | 613 (22.1) | 621 (22.4) | 164 (6.0) | 144 (5.9) | 299 (11.5) |
|  | Child of migrant(s) | (n = 170) | 104 (62.3) | 90 (53.9) | 51 (30.0) | 48 (28.2) | 31 (18.6) | 37 (21.8) | 39 (23.5) | 7 (4.2) | 10 (6.4) | 15 (9.4) |
|  | Migrant | (n = 120) | 79 (67.0) | 79 (65.8) | 53 (44.2) | 32 (26.9) | 35 (29.2) | 29 (24.4) | 39 (32.5) | 14 (11.9) | 6 (5.9) | 19 (16.5) |
|  | *Missing* |  | 119 | 94 | 70 | 60 | 43 | 50 | 36 | 13 | 8 | 18 |
| **Education level** | | |  |  |  |  |  |  |  |  |  |  |
|  | Low | (n = 966) | 665 (69.4) | 618 (64.5) | 390 (40.4) | 306 (31.8) | 275 (28.6) | 278 (28.9) | 301 (31.3) | 71 (7.5) | 67 (7.7) | 130 (14.5) |
|  | Middle | (n = 1384) | 863 (62.9) | 752 (54.7) | 461 (33.3) | 329 (23.9) | 274 (19.9) | 310 (22.5) | 295 (21.4) | 92 (6.7) | 70 (5.7) | 156 (11.9) |
|  | High | (n = 843) | 456 (54.3) | 382 (45.4) | 208 (24.7) | 135 (16.0) | 132 (15.7) | 132 (15.7) | 128 (15.3) | 30 (3.6) | 28 (3.8) | 63 (7.9) |
|  | *Missing* |  | 31 | 26 | 22 | 15 | 8 | 9 | 11 | 5 | 3 | 2 |
| **Employment status** | | |  |  |  |  |  |  |  |  |  |  |
|  | Employed ^*^ | (n = 1311) | 744 (56.9) | 659 (50.4) | 388 (29.6) | 275 (21.0) | 250 (19.1) | 253 (19.3) | 247 (18.9) | 80 (6.2) | 54 (4.7) | 144 (11.5) |
|  | Unemployed (or social benefit) | (n = 139) | 112 (81.8) | 97 (70.3) | 63 (45.3) | 39 (28.1) | 34 (24.5) | 36 (25.9) | 41 (29.5) | 20 (14.5) | 11 (9.0) | 22 (17.2) |
|  | Non-paid job^†^ | (n = 50) | 30 (60.0) | 26 (52.0) | 10 (20.0) | 12 (24.0) | 12 (24.0) | 12 (24.0) | 8 (16.0) | 2 (4.0) | 7 (16.7) | 4 (8.3) |
|  | Retired | (n = 1367) | 835 (61.8) | 731 (54.0) | 468 (34.2) | 332 (24.4) | 273 (20.1) | 309 (22.7) | 322 (23.7) | 68 (5.1) | 65 (5.3) | 118 (9.3) |
|  | *Missing* |  | 294 | 265 | 152 | 127 | 120 | 119 | 117 | 28 | 31 | 63 |
| **Net month income** (euro) | | |  |  |  |  |  |  |  |  |  |  |
|  | <1700 | (n = 1359) | 959 (71.3) | 860 (63.8) | 534 (39.3) | 407 (30.0) | 370 (27.4) | 365 (26.9) | 377 (27.9) | 110 (8.2) | 86 (7.1) | 183 (14.4) |
|  | 1700-2700 | (n = 957) | 556 (58.3) | 501 (52.5) | 289 (30.2) | 199 (20.8) | 162 (17.0) | 191 (20.0) | 201 (21.1) | 49 (5.2) | 37 (4.4) | 104 (11.4) |
|  | > 2700 | (n = 539) | 265 (49.3) | 214 (39.9) | 131 (24.3) | 78 (14.5) | 74 (13.8) | 77 (14.3) | 72 (13.4) | 19 (3.6) | 19 (4.0) | 30 (6.0) |
|  | *Missing* |  | 235 | 203 | 127 | 101 | 83 | 96 | 85 | 20 | 26 | 34 |
| **Household structure** | | |  |  |  |  |  |  |  |  |  |  |
|  | Alone | (n = 537) | 367 (69.1) | 341 (64.3) | 188 (35.0) | 135 (25.3) | 128 (23.9) | 138 (25.8) | 153 (28.7) | 38 (7.2) | 28 (5.9) | 57 (11.5) |
|  | With partner without children | (n = 1981) | 1204 (61.2) | 1042 (52.9) | 667 (33.7) | 472 (23.9) | 391 (19.9) | 432 (21.9) | 422 (21.4) | 99 (5.1) | 99 (5.6) | 188 (10.1) |
|  | With partner and children | (n = 477) | 290 (60.9) | 250 (52.5) | 142 (29.8) | 107 (22.5) | 109 (22.9) | 97 (20.4) | 98 (20.6) | 32 (6.8) | 21 (5.0) | 63 (13.9) |
|  | Together with someone else^‡^ | (n = 162) | 93 (58.1) | 92 (56.8) | 50 (30.9) | 40 (24.8) | 45 (27.8) | 36 (22.4) | 37 (22.8) | 17 (10.6) | 15 (11.4) | 34 (22.1) |
|  | Nursing home | (n = 29) | 23 (82.1) | 23 (79.3) | 10 (34.5) | 10 (34.5) | 4 (13.8) | 9 (31.0) | 9 (31.0) | 3 (10.7) | 3 (13.6) | 5 (19.2) |
|  | *Missing* |  | 38 | 30 | 24 | 21 | 12 | 17 | 16 | 9 | 2 | 4 |

Definition of abbreviations:

* Paid work, own company or living on one’s investments

† Such as homemaker

‡ Living together with someone else than partner, such as brother, parents or child.

Fatigue was defined by a score of >37 on the Checklist Individual Strength – fatigue subscale (CIS-8). Presence of new physical complaints was defined when patient reported one of the two last options of severity on the 4 point Likert scale (never, sometimes, often, almost always). Anxiety and depression symptoms were defined by a score of ≥ 8 on the Hospital Anxiety and Depression Scale (HADS) subscales, and Post-Traumatic Stress Disorder (PTSD) symptoms by a mean of all questions ≥ 1.75 on the Impact of Event Scale-6 (IES-6). Cognitive outcome was defined as cognitive impairment with a score of ≥43 on the abbreviated Cognitive Failure Questionnaire (CFQ-14).

# Supplemental 6.

Associations between social determinants of health and health problems one year after ICU admission (n = 3246) (extended version of Table 2 in the main text).

| Social determinants – N (%) | | Physical problems | | Psychological problems | | Cognitive problems | | Composite score^§^ | |
| --- | --- | --- | --- | --- | --- | --- | --- | --- | --- |
| Origin | | aOR (95% CI) | p | aOR (95% CI) | p | aOR (95% CI) | p | aOR (95% CI) | p |
|  | Country of origin: the Netherlands (R) | - |  | - |  | - |  | **-** |  |
|  | Child of migrant(s) | 0.89 (0.59-1.32) | .56 | 0.80 (0.50-1.25) | .33 | 0.74 (0.35-1.43) | .40 | 0.77 (0.50-1.19) | .25 |
|  | Migrant | 1.46 (0.93-2.28) | .10 | **2.03 (1.25-3.24)** | **<.01** | 1.18 (0.54-2.32) | .66 | 1.23 (0.75-2.01) | .42 |
| Education level | |  |  |  |  |  |  |  |  |
|  | High (R) | - |  | - |  | - |  | - |  |
|  | Middle | **1.26 (1.00-1.57)** | **.05** | 1.10 (0.84-1.43) | 0.49 | 1.28 (0.86-1.93) | .22 | 1.10 (0.86-1.39) | .48 |
|  | Low | **1.66 (1.28-2.16)** | **<.001** | **1.77 (1.32-2.38)** | **<.001** | 1.43 (0.91-2.26) | .12 | **1.84 (1.39-2.44)** | **<.001** |
| Employment status | |  |  |  |  |  |  |  |  |
|  | Employed (R) ^*^ | - |  | - |  | - |  | - |  |
|  | Unemployed (or social benefit) | **1.98 (1.31-3.01)** | **.001** | 1.18 (0.74-1.84) | .48 | 1.40 (0.74-2.51) | .28 | 1.46 (0.92-2.32) | .11 |
|  | Non-paid job^†^ | 0.63 (0.27-1.42) | .28 | **0.26 (0.08-0.73)** | **.02** | **0.11 (0.01-0.59)** | **.04** | 0.43 (0.16-1.09) | .08 |
|  | Retired | 1.13 (0.88-1.45) | .35 | 1.20 (0.90-1.61) | .21 | 0.80 (0.51-1.25) | .32 | 1.07 (0.81-1.41) | .63 |
| Net income (euro) | |  |  |  |  |  |  |  |  |
|  | > 2700 (R) | - |  | - |  | - |  | - |  |
|  | 1700-2700 | 1.10 (0.85-1.42) | .49 | **1.43 (1.05-1.95)** | **.02** | **1.79 (1.10-3.00)** | **.02** | 1.12 (0.85-1.47) | .44 |
|  | <1700 | 1.10 (0.83-1.47) | .51 | **1.54 (1.10-2.16)** | **.01** | 1.38 (0.80-2.43) | .25 | 1.20 (0.88-1.63) | .25 |
| Household structure | |  |  |  |  |  |  |  |  |
|  | Alone (R) | - |  | - |  | - |  | - |  |
|  | With partner without children | 0.98 (0.77-1.25) | .86 | 0.82 (0.63-1.08) | .16 | 0.82 (0.55-1.24) | .33 | 0.87 (0.67-1.13) | .30 |
|  | With partner and children | 1.03 (0.74-1.43) | .87 | 0.86 (0.59-1.25) | .44 | 1.17 (0.70-1.97) | .55 | 0.85 (0.60-1.22) | .38 |
|  | Together with someone else^‡^ | 1.28 (0.77-2.14) | .34 | 1.29 (0.73-2.25) | .37 | 1.74 (0.83-3.53) | .13 | 1.15 (0.65-2.06) | .63 |
|  | Nursing home | 0.80 (0.28-2.35) | .68 | 0.57 (0.18-1.70) | .33 | 1.72 (0.35-6.34) | .45 | 1.03 (0.31-4.00) | .97 |

Adjusted for: age, gender, admission type, Acute Physiology And Chronic Health Evaluation (APACHE) IV score and baseline score of the health variable (not applicable for new physical complaints and PTSD).

Definition of abbreviations: aOR = Adjusted Odds Ratio; CI = confidence interval; R = reference. Bold font indicates statistically significant difference (p < 0.05).

* Paid work, own company or living on one’s investments

† Such as homemaker

‡ Living together with someone else than partner, such as brother, parents or child.

^§^ Composite score of physical, psychological and cognitive health problems

# Supplemental 7.

Associations between several social determinants of health, and prevalence of physical and psychological health problems one year after ICU admission.

|  | | Physical | | | | Psychological | | | | | |
| --- | --- | --- | --- | --- | --- | --- | --- | --- | --- | --- | --- |
| Social determinants – N (%) | | Fatigue | | New physical complaints | | Anxiety | | Depression | | PTSD | |
| Origin – n/N (%) | | aOR (95% CI) | p | aOR (95% CI) | p | aOR (95% CI) | p | aOR (95% CI) | p | aOR (95% CI) | p |
|  | Country of origin: the Netherlands (R) | - |  | - |  | - |  | - |  | - |  |
|  | Child of migrant(s) | 1.00 (0.67-1.51) | .99 | 0.90 (0.59-1.34) | .60 | 0.65 (0.38-1.08) | .11 | 0.83 (0.49-1.35) | .46 | 0.71 (0.27-1.55) | .44 |
|  | Migrant | 1.53 (0.96-2.46) | .08 | 1.46 (0.94-2.24) | .09 | 1.41 (0.81-2.37) | .20 | **1.71 (1.00-2.83)** | **.04** | 1.95 (0.92-3.75) | .06 |
| Education level – n/N (%) | |  |  |  |  |  |  |  |  |  |  |
|  | High (R) | - |  | - |  | - |  | - |  | - |  |
|  | Middle | 1.25 (1.00-2.24) | .05 | **1.42 (1.12-1.79)** | **<.01** | 0.96 (0.72-1.30) | .80 | 1.31 (0.98-1.77) | .07 | **1.70 (1.06-2.79)** | **.03** |
|  | Low | **1.71 (1.31-2.24)** | **<.001** | **1.86 (1.43-2.42)** | **<.001** | **1.49 (1.08-2.07)** | .02 | **2.07 (1.50-2.87)** | **<.001** | **1.81 (1.06-3.15)** | **.03** |
| Employment status | |  |  |  |  |  |  |  |  |  |  |
|  | Employed (R) ^*^ | - |  | - |  | - |  | - |  | - |  |
|  | Unemployed (or social benefit) | 1.43 (0.93-2.23) | .11 | **1.84 (1.24-2.73)** | **<.01** | 1.01 (0.61-1.65) | .96 | 1.32 (0.80-2.12) | .27 | **2.19 (1.15-3.96)** | **.01** |
|  | Non-paid job^†^ | 0.62 (0.27-1.40) | .24 | 0.70 (0.27-1.64) | .44 | 0.55 (0.17-1.50) | .27 | 0.26 (0.06-0.88) | .05 | 0.35 (0.02-1.81) | .32 |
|  | Retired | 1.13 (0.88-1.46) | .35 | 1.01 (0.78-1.30) | .97 | 1.06 (0.77-1.47) | .71 | 1.17 (0.86-1.61) | .33 | 1.18 (0.71-1.98) | .54 |
| Net income (euro) | |  |  |  |  |  |  |  |  |  |  |
|  | > 2700 (R) | - |  | - |  | - |  | - |  | - |  |
|  | 1700-2700 | **1.36 (1.05-1.75)** | **.02** | 1.24 (0.93-1.67) | .26 | 1.09 (0.77-1.55) | .65 | 1.39 (0.99-1.98) | .06 | 1.11 (0.63-2.02) | .73 |
|  | <1700 | 1.29 (0.97-1.71) | .09 | 1.17 (0.89-1.53) | .15 | 1.44 (1.0-2.11) | .06 | 1.39 (0.96-2.04) | .08 | 1.17 (0.65-2.22) | .61 |
| Household structure | |  |  |  |  |  |  |  |  |  |  |
|  | Alone (R) | - |  | - |  | - |  | - |  | - |  |
|  | With partner without children | **0.70 (0.55-0.90)** | **<.01** | 1.13 (0.89-1.45) | .32 | 0.88 (0.65-1.19) | .39 | 0.82 (0.62-1.10) | .18 | 0.79 (0.51-1.25) | .30 |
|  | With partner and children | 0.77 (0.55-1.08) | .13 | 1.11 (0.79-1.55) | .56 | 1.01 (0.67-1.52) | .98 | 0.92 (0.61-1.39) | .71 | 0.75 (0.40-1.40) | .37 |
|  | Together with someone else^‡^ | 0.87 (0.52-1.48) | .61 | 1.12 (0.65-1.88) | .68 | 1.52 (0.84-2.72) | .16 | 1.04 (0.55-1.93) | .90 | 1.48 (0.64-3.22) | .34 |
|  | Nursing home | 1.92 (0.54-9.38) | .36 | 0.85 (0.28-2.34) | .76 | 0.26 (0.04-1.02) | .09 | 1.06 (0.31-3.23) | .93 | 2.14 (0.47-7.25) | .26 |

Adjusted for: age, gender, admission type, Acute Physiology And Chronic Health Evaluation (APACHE) IV score and baseline score of the health variable (not applicable for new physical complaints and PTSD).

Definition of abbreviations: aOR = Adjusted Odds Ratio; CI = confidence interval; R = reference. Bold font indicates statistically significant difference (p < .05).

* Paid work, own company or living on one’s investments

† Such as homemaker

‡ Living together with someone else than partner, such as brother, parents or child.
